# The Phenotypes and Functions of Neutrophils in Systemic Sclerosis

**DOI:** 10.3390/biom14091054

**Published:** 2024-08-25

**Authors:** Jiao Luo, Zhongming Xie, Lihua Duan

**Affiliations:** 1 Jiangxi Province Key Laboratory of Immunity and Inflammation, Jiangxi Provincial People’s Hospital, Nanchang 330000, China; luojiao@ncmc.edu.cn (J.L.); xiezhongming@ncmc.edu.cn (Z.X.); 2 Department of Rheumatology and Clinical Immunology, Jiangxi Provincial People’s Hospital, the First Affiliated Hospital of Nanchang Medical College, Nanchang 330000, China

**Keywords:** neutrophils, systemic sclerosis (SSc)

## Abstract

Systemic sclerosis (SSc) is a chronic disease of the connective tissue characterized by its multifaceted impact on various bodily systems, yet its precise cause remains elusive. Central to its pathology are abnormal immune activation, vasculopathy, and consequent fibrosis affecting both the skin and internal organs. The intricate interplay between the innate and adaptive immune systems significantly influences the pathogenesis of SSc. Despite substantial research, the role of neutrophils, key players in innate immunity, in the context of SSc has remained enigmatic. Emerging evidence suggests that neutrophils not only contribute to the initiation and perpetuation of SSc but also inflict damage on organs and promote fibrosis—a hallmark of the disease in many patients. This review aims to investigate the nuanced involvement of neutrophils in the development of SSc. By shedding light on the intricate mechanisms through which neutrophils influence the pathogenesis of SSc, we can gain deeper insights into the disease process and potentially identify novel therapeutic targets. Understanding the precise role of neutrophils may pave the way for more targeted and effective interventions to alleviate the burden of SSc on affected individuals.

## 1. Introduction

Systemic sclerosis (SSc) and scleroderma represent autoimmune disorders marked by severe clinical manifestations and heightened mortality rates. Unfortunately, current treatment approaches for this disease remain limited and only partially effective [1]. SSc is characterized by vasculopathy, digital ulcers, Raynaud’s phenomenon, renal failure, pulmonary arterial hypertension, and fibrosis [2,3]. Its primary association with reduced survival stems from pulmonary fibrosis (interstitial lung disease) and pulmonary arterial hypertension [4]. Two subgroups of SSc are defined by the extent of skin involvement. The skin thickening of limited cutaneous SSc (LcSSc) predominantly involves the hands, arms, and face, whereas diffuse cutaneous disease (DcSSc) involves more extensive skin thickening [5,6]. SSc is found in approximately 150 to 300 individuals out of every 1,000,000 [7]. Females carry a higher risk than males. Moreover, genetic factors play a significant role. The occurrence of SSc in families with a history of the disease varies from 0.026% to 1.5–1.7% [8]. First-degree relatives are also predisposed to Raynaud’s phenomenon (RP) and other autoimmune diseases (AIDs) [9]. Genes encoding proteins regulating innate immunity, macrophage activation, and T cell functions are implicated [10,11]. In addition, DNA methylation, histone modifications, and non-coding RNAs significantly influence disease characteristics in systemic sclerosis (SSc). Notably, histone modifications play a pivotal role in determining the fate of these disease features. DNA methyltransferases are essential in the pathogenesis of the disease, as they mediate the methylation of DNA at specific promoter regions, thereby regulating the expression of particular pathways [12]. Besides the impact of genetic predispositions on SSc, the clinical and pathologic expressions of SSc arise from abnormalities in the innate/adaptive immune system, which cause the generation of autoantibodies and cell-mediated autoimmunity, respectively. Furthermore, the skin and internal organs experience an excessive buildup of collagen and other matrix components due to microvascular endothelial cells, fibroproliferative vasculopathy in small vessels, and dysfunction of fibroblasts [13].

Polymorphonuclear leukocytes (PMNs), primarily neutrophils, are the most abundant white blood cells in the human bloodstream. After maturing in the bone marrow, neutrophils enter the bloodstream, where their half-life is estimated to be only 5–10 h [14]. Despite their short lifespan, neutrophils, which make up the initial defense against pathogens, are now recognized as significant elements in the effector and regulatory circuits of both the innate and adaptive immune systems. Neutrophils, as core components of innate immunity, are essential in protecting against bacterial infections. They detect damage-associated molecular patterns (DAMPs) and pathogen-associated molecular patterns (PAMPs), such as lipopolysaccharide (LPS) or peptidoglycan [15]. Furthermore, neutrophils function by means of phagocytosis, respiratory burst, degranulation, the discharge of reactive oxygen species (ROS), the creation of neutrophil extracellular traps (NETs), and the emission of cytolytic enzymes. Given the pivotal role of neutrophils in immune responses, their involvement in the development of autoimmune diseases should be reassessed. In this context, we explore the involvement of neutrophils in SSc.

## 2. Classification of Neutrophils

Neutrophils are derived from the common myeloid progenitor (CMP) and persist in extramedullary organs like the spleen through the assistance of granulocyte colony-stimulating factor (G-CSF) [16]. Neutrophil development can be roughly categorized into two phases: an initial stage involving the growth of promyelocytes and bone marrow cells, and a subsequent stage where the neutrophils progress from band cells to fully developed segmented neutrophils, with no further proliferation [17]. In general, the different phases of granulocyte creation are described based on cell dimensions, nucleus clustering, and particle composition, which might not precisely represent their operational characteristics or effectively differentiate between distinct phases of neutrophil growth. Based on varying levels of CD101, CD49d, CD10, CD15, CD16, and CD11b surface expression, advances in single-cell transcriptomics and mass cytology have revealed three distinct developmental stages of post-mitotic neutrophils in human bone marrow: precursor, immature, and mature neutrophils [18]. Typically, neutrophil production primarily occurs in the bone marrow. Emergency granulocytes may be produced in certain specific situations, like severe infections or autoimmune disorders, to expedite the growth and development of neutrophil precursor cells while decreasing the production of lymphocytes and monocytes [19]. The rapid mobilization of mature neutrophils can also lead to the presence of peripheral immature neutrophils, which may arise from emergent granulopoiesis. The movement of these undeveloped neutrophils to the outer edges suggests that inflammation in the surrounding tissue can impact the characteristics of neutrophils in the bloodstream, leading to the development of modified subtypes of neutrophils with different functional and phenotypic attributes in various autoimmune disorders [20]. In other words, neutrophils are not simply a uniform group of cells but rather a multifaceted cell category with different subtypes that vary in terms of their characteristics and functions. In 1986, low-density granulocytes (LDG) were initially discovered in individuals with systemic lupus erythematosus, rheumatoid arthritis, and acute rheumatic fever, thus constituting different subtypes of neutrophils. Low-density neutrophils, which are present in higher quantities in systemic lupus erythematosus, vasculitis associated with antineutrocytic cytoplasmic antibodies, psoriasis, and various inflammatory conditions, exhibit an increased capacity to generate immunogenic nets, leading to greater tissue damage and possessing higher immunostimulatory and invasive properties compared to nets produced by other subsets of neutrophils [21,22,23,24]. In cancer patients, there exists another type of neutrophil known as polymorphonuclear-myeloid-derived suppressor cells (PMN-MDSC) that possess notable immunosuppressive properties. Typically, antineoplastic neutrophils can eliminate tumor cells by discharging reactive oxygen species (ROS) and reactive nitrogen species (RNS) [25]. Additionally, they stimulate T cell activation and attract pro-inflammatory (M1) macrophages. In contrast, certain tumor neutrophils have the ability to secrete matrix metalloproteinase 9 (MMP9), thereby facilitating the angiogenesis and spread of tumor cells. They also impede the functionality of natural killer (NK) cells. Furthermore, they have the ability to enlist anti-inflammatory (M2) macrophages and T regulatory cells. Moreover, it can inhibit the function of CD8^+^T cells [26]. In individuals with sepsis, a distinct form of neutrophil has been identified, displaying notable diversity, impairment, and immune suppression. This variant hinders T cell activity and prompts T cell transdifferentiation by means of the PD-L1 pathway [26].

## 3. The Function of Neutrophils

Circulating neutrophils are well known to have a feedback loop that decreases the expression of L-selectin (CD62L), increases the expression of CXCR4 and CD11b, and leads to nuclear hypersegmentation as neutrophils age and reach their peak every 24 h. Neutrophils with high levels of CXCR4 migrate to the bone marrow, where they are engulfed by nearby macrophages and substituted with neutrophils with high levels of CD62L and low levels of CXCR4, which then migrate to a different new peak [27]. Initially, neutrophils near the site of damage move towards the core. Subsequently, Neutrophils located at the core of the response generate leukotriene B4, initiating the further movement of neutrophils. Additionally, G protein-coupled receptors facilitate the gathering of the clustered neutrophils at the site of injury [28]. Pathogens activate neutrophils through the recognition of patterns by receptors like Toll-like receptors (TLRs) or opsonic receptors. This activation triggers the NADPH oxidase located on the plasma membrane to generate oxygen radicals and, ultimately, a cascade of reactive oxygen species (ROS), granule enzymes, and cytokines. Simultaneously, the activation of Ion pumps has also occurred, leading to the entry of ions like H^+^ and K^+^ [29]. The resolution of inflammation requires the effective apoptosis of neutrophils. Delayed apoptosis of neutrophils results in tissue damage and persistent inflammation [30].

## 4. The Function of Neutrophil Extracellular Traps (NETs)

Neutrophils have an alternative way of eliminating extracellular organisms through the discharge of neutrophil extracellular traps (NETs). These NETs consist of DNA and histones combined with granule enzymes like myeloperoxidase, elastase, lactoferrin, MMP-9, and Cytoplasmic protein LL37. They function as antimicrobial agents in pathogen-triggered NETosis but could potentially become autoantigens in autoimmune conditions. Activation of NETosis occurs through the engagement of certain receptors by bacteria, fungi, viruses, immune complexes, and crystals, leading to the involvement of diverse downstream effector proteins. The effective process of NETosis necessitates the involvement of histone citrullination, elastase 80, a serine protease, MPO127, and the NADPH oxidase complex [31,32]. Activation of neutrophil elastase (NE) is triggered by the myeloperoxidase (MPO) pathway, which is mediated by the induction of reactive oxygen species (ROS) via mitogen-activated protein kinases (MAPK)–extracellular-signal-regulated kinase (ERK) signaling. To hinder phagocytosis, NE breaks down the actin cytoskeleton in the cytoplasm and then moves to the nucleus where it promotes chromatin decondensation through histone processing. Chromatin decondensation occurs through MPO and the activation of protein-arginine deiminase type 4 (PAD4), which causes histone citrullination. Additionally, autophagy plays a role in the formation of NETs. By promoting the formation of phagosomes that sequester NE away from the nucleus, Dectin 1, acting as a phagocytic receptor, can hinder NETosis. The activation and generation of ROS by neutrophils are limited by Siglec-5 and Siglec-9, which also suppress NETosis. The inhibition of NE by endogenous serpin protease inhibitors prevents NETosis. The antimicrobial effects of NETs are counteracted by the action of the DNA-degrading enzyme, deoxyribonuclease 1 (DNase 1) [33,34]. NETs act as a framework that enhances the occurrence of deep vein thrombosis (DVT) in the bloodstream while also directly or indirectly controlling inflammatory cytokines by influencing other immune cells. NETs actively contribute to cellular and tissue harm in cases of infection, sepsis, autoimmunity, and diabetes [35].

## 5. The Function of Low-Density Granulocytes (LDG)

The identification of low-density neutrophils occurred in 1986 when neutrophils were isolated from the peripheral blood mononuclear cell group of individuals diagnosed with systemic lupus erythematosus (SLE) [36]. The pro-inflammatory nature of LDG in SLE has been reported due to their ability to release tumor necrosis factor-alpha (TNF-α), interferon gamma (IFN)-γ, and type I IFN. These cytokines are often associated with disease pathogenesis [37]. LDG plays a direct role in causing dysfunction in endothelial cells and damage to blood vessels. Additionally, LDG generates NETs, which have the ability to stimulate the production of type I IFN [21,33]. A higher incidence of skin involvement, vasculitis, arterial inflammation, and coronary atherosclerosis was observed to be associated with an increase in circulating LDG numbers [37,38]. LDGs also spontaneously release NETs in systemic lupus erythematosus (SLE) and RA, among various autoimmune conditions [39]. The combination of LDGs and deficiencies in NET clearance could lead to the extended presence of antigenic substances and the subsequent stimulation of proinflammatory cytokines, which may contribute to a continuous cycle that prolongs autoimmunity [40].

## 6. SSc Involves the Presence of Neutrophils and Autoantibodies

Anti-neutrophil cytoplasmic antibodies (ANCA) are a group of autoantibodies that cause systemic vascular inflammation by binding to target antigens of neutrophils. A positive staining of anti-neutrophil cytoplasmic antibodies (ANCA) on neutrophils can typically be categorized into three primary patterns using indirect immunofluorescence (IIF) on ethanol-fixed neutrophils. The first pattern, known as the cytoplasmic pattern (c-ANCA), is characterized by fluorescence staining within the cytoplasm of neutrophils. This pattern is predominantly associated with the presence of autoantibodies targeting serine proteinase-3 (PR3). The second pattern, known as perinuclear anti-neutrophil cytoplasmic antibody (p-ANCA) staining, is characterized by fluorescence surrounding the multi-lobed nucleus of neutrophils. This staining pattern is predominantly attributable to autoantibodies targeting myeloperoxidase (MPO). Lastly, atypical ANCA (a-ANCA) exhibits distinct patterns or a combination of both c-ANCA and p-ANCA patterns [41]. Activation of neutrophils can occur through ANCA-IgG via TNF-α, leading to the induction of neutrophil expression of the autoantigens MPO and PR3. These autoantigens persistently bind to endothelial cells, resulting in direct apoptosis [42]. While the occurrence of ANCA in the blood of individuals with SSc is not common, up to 12% of patients diagnosed with SSc possess ANCA. Jayneet reported that ANCA was linked to a higher occurrence of interstitial lung disease (ILD) and pulmonary embolism (PE) in SSc. ANCA identified individuals with more severe prognoses who needed careful monitoring for negative outcomes [43]. Rho et al. reported in 63 cases of ANCA-associated vasculitis (AAV) in SSc, it was discovered that 72% had a positive ANA (titers unknown), 70% had the anti-Scl-70 antibody, and 72% had a positive anti-MPO antibody [44]. Another eleven cases of AAV in SSc were reported and 82% of cases had renal involvement in the form of crescentic glomerulonephritis and 91% of cases had positive anti-MPO antibodies. The results indicated the significance of recognizing crescentic glomerulonephritis associated with AAV as a potential factor contributing to renal insufficiency in SSc patients [45].

Except for ANCA antibodies in SSc, other functional antibodies contacting extracellular receptors and inducing intracellular signaling pathway activation are also characteristics of SSc. Neutrophils play a role in the development of SSc through the involvement of these antibodies. Anti-endothelial cell antibodies (AECA) and anti-fibroblast antibodies (AFA) control the presence of adhesion molecules like E-selectin, ICAM-1, and VCAM-1 on endothelial cells, as stated in reference [46]. ICAM-1 also expresses on the neutrophils that result in accumulation and enhanced motility of transmigrated PMN in tissues [47]. Anti-angiotensin Ⅱ type 1 receptor (AT1R) and endothelin-1 type A receptor (ETAR) antibodies were found in patients with SSc [48]. AT1R and ETAR, present in both immune cells (neutrophils) and non-immune cells (endothelial cells and fibroblasts), control the response of fibroblasts, production of cytokines, and the vasoconstrictor impact on endothelial cells. Angiotensin II and endothelin-1 directly stimulate the movement of myeloid cells, including neutrophils [49,50]. The anti-AT1R and ETAR antibodies can cause the induction of IL-8 and ACAM1, resulting in enhanced homing and migration of neutrophils to the site of inflammation [51]. Studies suggest that the ET-1 system contributes to inflammation and fibrosis development in SSc by activating T lymphocytes and neutrophils, promoting cytokine release [52]. Fibroblasts and neutrophils also contribute to the generation of reactive oxygen species through the involvement of autoantibodies against AT1R and ETAR. Elevated serum antibody levels against ERα, identified in SSc patients, were associated with diffuse cutaneous SSc (dcSSc) activity. ER presence in neutrophils, NK cells, and other cells of the adaptive immune system that control the levels and activities of neutrophils such as chemotaxis, production of ROS and cytokines, as well as dendritic cell maturation, B and T lymphocyte differentiation [53,54].

## 7. Neutrophils and Inflammation in SSc

Neutrophils are integral to innate immunity, performing functions like phagocytosis, respiratory bursts, degranulation, ROS release, NET formation, and the secretion of cytolytic enzymes, cytokines, and chemokines. These actions help defense against bacterial infections. Moreover, neutrophils infiltrated and activated pro-inflammatory cytokines (IL-1a, IL-1ß, tumor necrosis factor (TNF), IL-6) at the location of tissue injury and pathogen penetration [55]. Neutrophils attract monocytes/macrophages and trigger adaptive reactions. In systemic sclerosis (SSc), elevated levels of inflammatory cytokines induce the formation of neutrophil extracellular trap (NET) by attracting neutrophils. Runa Kuley et al. discovered increased amounts of NETs (MPO-DNA complexes) in plasma samples obtained from individuals with SSc [56] and Daniela Impellizzieri et al. found that freshly isolated neutrophils from SSc patients had a severe defect in the formation of neutrophil extracellular traps [57]. This suggests that the inflammatory capacity of neutrophils in SSC patients may be severely depleted. Norma Maugeri et al. [58] found that microparticles released from activated platelets expressing HMGB1 were discovered in large quantities in the bloodstream of patients with SSc. These microparticles led to neutrophil activation and the production of NET. The presence of BoxA, a competitive inhibitor of HMGB1, further eliminated the production of NETs and neutrophil activation induced by these microparticles. This suggests that neutrophil activation is mediated by the activation of platelet microparticles by HMGB1. The induction of pro-fibrotic processes in scleroderma has been associated with the generation of a large amount of ROS, suggesting that ROS stress plays a role in this condition. The generation of reactive oxygen species is essential for the formation of neutrophil extracellular traps, the processes of neutrophil death, and the identification of well-established indicators in conditions like rheumatoid arthritis and systemic lupus erythematosus. Neutrophils additionally aid in the progression of wound healing by facilitating the formation of alternatively activated macrophages, which release anti-inflammatory mediators such as IL-4, IL-13, and IL-10. A study demonstrated that reducing neutrophil levels accelerates wound healing in rats, underscoring their crucial role in the initial inflammatory response [59]. Typically, the quantity of neutrophils at a site of inflammation is generally minimal due to their short lifespan and rapid turnover rate. Investigating the rate of neutrophil replacement at these sites and their interactions with macrophages and dendritic cells is imperative, especially in cases of chronic inflammation and fibrosis. IL-36 as part of the larger IL-1 family of cytokines includes three agonists, IL-36α, IL-36β, and IL-36γ, and two inhibitors, IL-36RA and IL-38. IL-36α undergoes proteolytic cleavage predominantly facilitated by neutrophil-derived proteases, resulting in the generation of its biologically active cytokine form [60]. Steven found that serum IL-36 levels were significantly elevated in comparison to healthy controls. Additionally, neutrophil elastase levels were also elevated in the matched sera. Although IL-36 did not exhibit direct pro-fibrotic effects on dermal fibroblasts, it did induce the release of pro-inflammatory cytokines, a process dependent on the MAPK pathway. Furthermore, IL-36α stimulated the release of CCL20 and CCL2 in keratinocytes, which may contribute to fibrosis [61]. It is suggested that, while IL-36α may enhance fibrosis through keratinocyte–immune fibroblast cross-talk. Whether the elevation of IL-36α is due to the activation of neutrophils needs further investigation.

## 8. Neutrophils and Vasculopathy and Fibrosis in SSc

In systemic sclerosis (SSc), initial vascular damage is caused by environmental factors, autoimmune attacks from anti-endothelial cell antibodies (AECAs) and γδT cells. This damage then leads to structural and functional abnormalities, ultimately resulting in tissue fibrosis [62]. Neda Kortam et al. found that neutrophil activation and the release of neutrophil extracellular traps (NETs) were significantly elevated in patients with systemic sclerosis (SSc)-associated vascular complications compared to matched patients without such complications. Furthermore, neutrophil activation and NETs exhibited a positive correlation with soluble E-selectin and vascular cell adhesion molecule-1 (VCAM-1), both of which are circulating markers indicative of vascular injury. The administration of a synthetic prostacyclin analog to patients experiencing digital ischemia resulted in an increase in neutrophil cyclic adenosine monophosphate (cAMP) levels, which was associated with a reduction in NET release and a subsequent decrease in circulating NETs [63]. This suggests a link between NETs and vascular complications in SSc. Liya and colleagues showed that exosomes derived from neutrophils in individuals with systemic sclerosis can suppress the growth and movement of endothelial cells; alveolar epithelial cells were damaged and the permeability of alveolar and capillary vessels was increased by neutrophils [64]. Endothelial cells that are stimulated and express adhesion molecules, specifically the selectins, have the ability to capture neutrophils from the bloodstream. The ß2 integrin family of proteins facilitates the rolling of neutrophils along the endothelium in this procedure. Neutrophils secrete pro-fibrotic cytokines like TGFß, IL-6, and VEGF [65]. Platelet activation can occur through the release of damp-associated molecular patterns (DAMPs) from endothelial cells that have been damaged, immune complexes, or complement effector molecules. Activated platelets additionally contribute to the inflammatory atmosphere and interact with endothelium and leukocytes by releasing immunoreactive substances and microparticles. These microparticles, abundant in the bloodstream of individuals with systemic sclerosis (SSc), exhibit increased proteolytic function, extended lifespan, and the ability to promote the formation of neutrophil extracellular traps (NETs). SSSc microparticles interact with neutrophils and promote neutrophil autophagy in vitro and in the immunocompromised mice model [58]. NETs directly kill epithelial and endothelial cells [66]. In addition, neutrophils that are activated release reactive oxygen species (ROS) [67]. Activated neutrophils release reactive oxygen species (ROS), which play a significant role in fibrosis by activating fibroblasts and stimulating the release of TNFβ [68,69]. The excessive ROS generated by NETs have a significant impact on fibrosis, as ROS react with latent TGFβ, disrupting its structure and activating it. Studies have shown the presence of α-SMA positive myofibroblasts in fibrotic skin, lung, and skin biopsy samples from SSc patients, confirming the role of NETs in fibrosis [70]. SSc serum triggers activation and apoptosis of endothelial cells in co-cultures with neutrophils, primarily through IL-6 and reliance on neutrophil presence [71]. Furthermore, SSc neutrophils are predisposed to generating ROS and can bind to activated endothelium, sustaining endothelial dysfunction and facilitating fibrosis [67]. The primary source of reactive oxygen species (ROS) generation within cells is the stimulation of the NADPH oxidase pathway or NOX enzyme. Decreasing or eliminating the impact of NOX4 reduces the generation of ROS and the expression of genes that encode fibrotic proteins. Neutrophil elastase also triggers the development of myofibroblasts; mice lacking this enzyme were shielded from lung fibrosis caused by asbestos. In vitro, neutrophil elastase induced lung fibroblast proliferation, myofibroblast differentiation, and contractility [72]. Sivelestat, a pharmacological inhibitor of neutrophil elastase, shielded mice from lung fibrosis caused by bleomycin [73]. According to Crestani B’s research, the entry of neutrophils into the alveolus, which can be triggered by IL-8 discharged by alveolar macrophages, may contribute to the development of SSc lung disease by releasing elastase [74]. In SSc, Nancy Wareing highlighted the prognostic importance of blood neutrophil count and the neutrophil-to-lymphocyte ratio (NLR) in relation to disease severity and mortality. Higher neutrophil count and NLR predict a more severe disease course and higher mortality [75]. As is well known, IL-1 represents a quintessential pro-inflammatory cytokine. The designation IL-1 encompasses two distinct molecules, IL-1α and IL-1β, which exhibit substantial sequence homology and engage the same IL-1 type-I receptor (IL-1RI). This receptor subsequently transduces pro-inflammatory signals, culminating in the synthesis and expression of numerous secondary inflammatory mediators. IL-1α regulates the differentiation of fibroblasts into myofibroblasts and influences the longevity of myofibroblasts, both of which are pivotal processes in systemic sclerosis (SSc) [76]. However, endogenous IL-1α stimulates fibroblast proliferation and collagen synthesis through the induction of interleukin-6 (IL-6) and platelet-derived growth factor (PDGF) [77]. In addition, elevated levels of interleukin-1 beta (IL-1β) are detectable in both bronchoalveolar lavage fluid (BAL) and serum in the patients with SSc [78]. Additionally, in the affected skin of SSc patients, IL-1β and interleukin-18 (IL-18) are significantly overexpressed, a phenomenon that correlates with the extent of skin fibrosis as measured by the modified Rodnan skin score (mRSS) [79]. IL-1β also promotes myofibroblast activation, endothelial-to-mesenchymal transition, and fibrosis through the mediation of interleukin-6 (IL-6) and transforming growth factor-beta (TGF-β1) [80]. Based on this, whether neutrophils participate in the fibrotic mechanism of SSc by secreting IL1β deserves to be investigated.

In the lungs, the extracellular matrix (ECM) constitutes a highly organized and intricate network structure, comprising macromolecules such as proteins and polysaccharides that are synthesized by cells and subsequently secreted into the extracellular milieu. The ECM includes components such as collagen, elastin, fibronectin, laminin, glycosaminoglycans, and proteoglycans [81,82]. The interstitial matrix, a subset of the ECM, is characterized by a loose arrangement of collagen fibers, predominantly composed of type I and type III collagen, fibronectin, elastin, and various proteoglycans. Neutrophils contribute to extracellular matrix (ECM) remodeling through the secretion of specific matrix-remodeling enzymes, including neutrophil elastase and metalloproteinases, the formation of neutrophil extracellular traps, and the release of exosomes. Conversely, the ECM can modulate the inflammatory microenvironment by influencing neutrophil function, thereby driving disease progression. The presence of the ECM and the dynamic interplay between neutrophils and their extracellular matrices represent a critical mechanistic aspect of inflammation [83].

## 9. Conclusions

**Figure 1:**
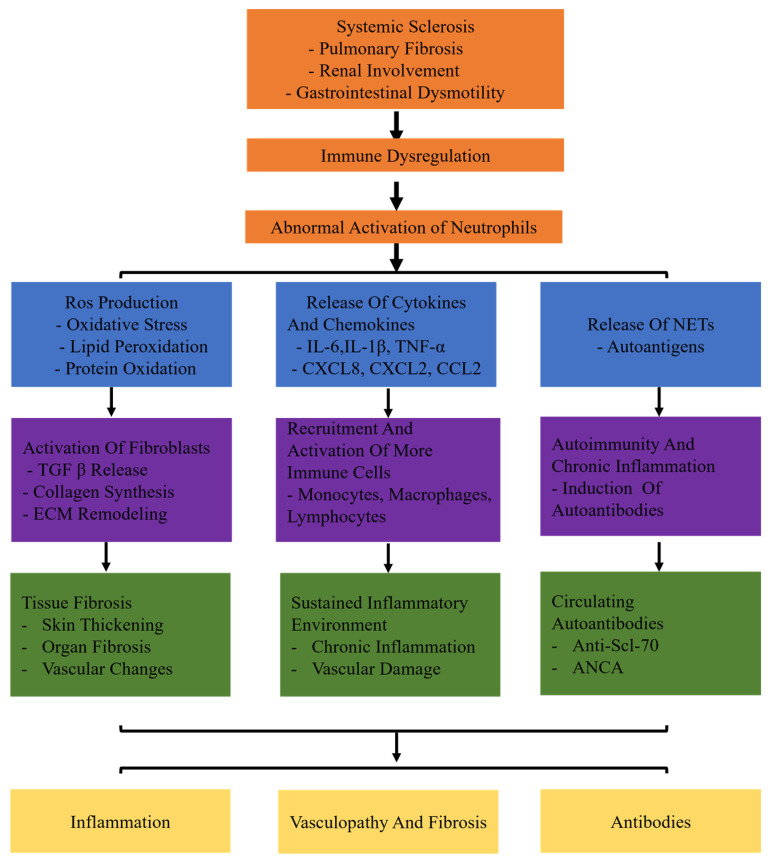
The specific mechanisms of neutrophils in systemic sclerosis (SSc). Systemic sclerosis leads to immune dysregulation, causing abnormal activation of neutrophils. Abnormally activated neutrophils release ROS and proteolytic enzymes. Production of ROS leads to oxidative stress, lipid peroxidation, and protein oxidation. Release of cytokines (IL-6, TNF-α, IL-1β) and chemokines (CXCL8, CXCL2, CCL2) triggers inflammatory responses. Release of neutrophil extracellular traps (NETs) induces autoimmunity and chronic inflammation. Neutrophils release TGF-β, activate fibroblasts, and promote collagen synthesis and extracellular matrix (ECM) remodeling. In addition, neutrophils recruit and activate more immune cells (monocytes, macrophages, lymphocytes). It results in chronic inflammation and vascular damage persists. Moreover, neutrophils induce the production of autoantibodies.

Recent evidence suggests that neutrophil activation plays a significant role in the development of SSc. However, the specific mechanism of an abnormal subset of neutrophils has been unclear so far. This review aims to elucidate the involvement of neutrophils in SSc. In systemic sclerosis (SSc), neutrophils are abnormally activated due to immune dysregulation. These activated neutrophils contribute to the disease through three main pathways: inflammation, fibrosis, and autoantibody production. In the inflammatory pathway, neutrophils release reactive oxygen species (ROS) and cytokines such as IL-6, TNF-α, and MMP-9, which lead to tissue damage and remodeling. In the fibrotic pathway, neutrophils promote the deposition of extracellular matrix and collagen production through factors like TGF-β and PDGF, driving myofibroblast differentiation. Additionally, neutrophil-derived antigens, including components of neutrophil extracellular traps (NETs), induce autoimmune responses and activate adaptive immune cells, contributing to the production of autoantibodies. These combined mechanisms of inflammation, fibrosis, and autoimmunity illustrate the multifaceted role of neutrophils in the pathogenesis of systemic sclerosis (Figure 1). Gaining a comprehensive comprehension of the function of neutrophils in the inflammatory and fibrosis processes of SSc will aid in advancing novel and efficient therapies to hinder the advancement of SSc.
